# RSV A2-Based Prefusion F Vaccine Candidates Induce RSV A and RSV B Cross Binding and Neutralizing Antibodies and Provide Protection against RSV A and RSV B Challenge in Preclinical Models

**DOI:** 10.3390/vaccines11030672

**Published:** 2023-03-16

**Authors:** Freek Cox, Eirikur Saeland, Anne Thoma, Ward van den Hoogen, Lisanne Tettero, Joke Drijver, Cornelis Vaneman, Yolinda van Polanen, Tina Ritschel, Arangassery Rosemary Bastian, Benoit Callendret, Roland Zahn, Leslie van der Fits

**Affiliations:** Janssen Vaccines & Prevention B.V. Archimedesweg 4-6, 2333 CN Leiden, The Netherlands

**Keywords:** Adenoviral vector 26, cotton rat model, respiratory syncytial virus, RSV prefusion protein, RSV subtypes

## Abstract

RSV is divided into two antigenic subtypes, RSV A and RSV B, which is largely based on the variation in the G protein, while the fusion protein F is more conserved and a target for antibody-mediated neutralization. Here we evaluate the breadth of the protective immune responses across RSV A and RSV B subtypes, induced by vaccines based on the RSV A-based fusion protein, stabilized in the prefusion conformation (preF) in preclinical models. Immunization of naïve cotton rats with preF subunit or preF encoded by a replication incompetent Adenoviral 26, induced antibodies capable of neutralizing recent RSV A and RSV B clinical isolates, as well as protective efficacy against a challenge with RSV A and RSV B strains. Similarly, induction of cross-neutralizing antibodies was observed after immunization with Ad26-encoded preF, preF protein or a mix of both (Ad26/preF protein) in RSV pre-exposed mice and African Green Monkeys. Transfer of serum of human subjects immunized with Ad26/preF protein into cotton rats provide protection against challenges with both RSV A and RSV B, with complete protection against both strains observed in the lower respiratory tract. In contrast, almost no protection against RSV A and B infection was observed after the transfer of a human serum pool isolated pre-vaccination. These results collectively show that the RSV A-based monovalent Ad26/preF protein vaccine induced neutralizing antibodies, as well as protection against both RSV A and RSV B subtypes in animals, including by passive transfer of human antibodies alone, suggesting that clinical efficacy against both subtypes can be achieved.

## 1. Introduction

Respiratory syncytial virus (RSV) is a single-stranded, negative-strand RSV virus that belongs to the family *Pneumoviridae*. In healthy adults, RSV infection is usually mild and manifests itself with common cold-like symptoms that resolve on their own. However, RSV is an increasingly recognized cause of severe lower respiratory tract disease in the elderly or adults with underlying medical conditions. It has been shown that RSV causes higher morbidity and mortality in older hospitalized adults compared to influenza, with possibly an even greater impact on long-term survival [1]. A recent study describes an estimated ~1.5 million episodes of RSV-associated acute respiratory infection in older adults in 2015 for industrialized countries globally, of which about 14.5% of the affected subjects were hospitalized, resulting in an estimated 14.000 in-hospital deaths related to RSV globally [2]. Currently, supportive care is the main treatment option for RSV patients in all age groups. The only approved immunoprophylactic products are monoclonal antibodies (MAbs) intended for pediatric use, such as Palivizumab and Nirsevimab.

Virus-neutralizing antibodies have been shown to be important for protection against RSV infection and disease [3,4]. The RSV fusion (F) protein is the major target for antibody-mediated neutralization and is also the target of the prophylactic MAbs Palivizumab and Nirsevimab. The F protein exists in two different conformational states; it transitions from the metastable prefusion (preF) into the highly stable post-fusion (postF) conformation during the fusion of the virus and target cells. Most potent F protein-directed neutralizing antibodies in the serum of RSV-positive individuals are directed against the preF conformation [5]. In the past, vaccine candidates were developed based on the F protein sequence. However, due to the metastable nature of the preF protein, those were mainly in the postF conformation, known to be a relatively poor inducer of neutralizing antibodies [6,7]. Consequently, two postF-like vaccine candidates have failed in human Phase 3 trials [8,9]. Recently, different structural design-based approaches resulted in the stabilization of the F protein in its prefusion conformation. When compared to postF, stabilized preF was shown to elicit higher protective immunity, accompanied by higher levels of neutralizing antibodies in preclinical models [10,11]. Multiple preF-based vaccine candidates are currently in Phase 3 clinical testing for protective efficacy in humans, and the first results announced indicate promising efficacy. 

RSV is divided into two major antigenic subtypes, RSV A and RSV B, that co-circulate during seasonal epidemics, often with a predominance of one of the subtypes in a single season. Sequence analysis indicated that multiple clades for both RSV A and RSV B can co-circulate globally, with the same predominant clades of viruses being found in countries around the world, suggesting that the evolution of RSV is not strongly regionalized [12]. Several studies examined the relationship between RSV genotype and clinical severity; however, due to conflicting results, these have failed to reach a consensus [13]. The classification is largely defined by the genetic variation in the attachment glycoprotein G. Whereas the overall amino acid sequence identity between RSV A and RSV B for the G protein is limited to approximately 53%, F proteins from RSV A and RSV B subtypes are highly similar, and share 89% amino acid identity [14]. Consequently, most antibodies directed against F are likely to be cross-reactive between the RSV subtypes. The RSV F-directed MAbs Palivizumab and Nirsevimab have shown *in vitro* neutralizing capability and protective efficacy in the cotton rat model against both RSV A and RSV B strains to a similar level [15]. In addition, similar clinical efficacy against both RSV A and RSV B subtypes was demonstrated for Palivizumab [16]. In contrast, the RSV subtype specificity of RSV F-directed monoclonal antibodies has been described in the literature; reduced binding of RSV F MAb D25 to preF derived from RSV B strains was demonstrated in [17,18], although similar RSV A and RSV B neutralization capacity for this antibody was shown by others [19]. Recently, an RSV F MAb, Suptavumab (REGN2222), failed in a Phase III clinical efficacy study. Whereas 60% efficacy was observed against infection with RSV A strains, no efficacy was observed for RSV B strains [20]. In contrast to MAb, vaccines induce polyclonal antibody responses that are likely to be more broadly neutralizing and less vulnerable for viral escape mutations or differences between RSV subtypes. However, after vaccination with the RSV A-based preF protein DS-Cav1, RSV subtype-specific skewing was demonstrated in preclinical and clinical studies [11,21], whereas this was not observed in mouse studies using an RSV B 18537-based DS-Cav1 preF protein [17]. 

We previously demonstrated that preF vaccine candidates based on RSV A2, either administered as soluble protein, encoded by a replication incompetent Adenoviral vector (Ad26.RSV.preF) or a combination of both, are immunogenic and can induce protection against infection with RSV A2 in the cotton rat challenge model [22,23]. Moreover, Ad26.RSV.preF provided significant protection in humans upon a challenge with RSV A Memphis 37 [24]. Studies described in this manuscript are conducted to assess the breadth of the immune responses induced by these RSV A2-based vaccine components by examining the induction of neutralizing antibodies and protection across RSV subtypes.

## 2. Materials and Methods

### 2.1. Ethics Statement

Mouse studies were conducted at Janssen Vaccines & Prevention B.V. according to the Dutch Animal Experimentation Act and the Guidelines on the Protection of Animals for scientific purposes by the Council of the European Committee after approval by the Centrale Commissie Dierproeven and the Dier Experimenten Commissie of Janssen Vaccines & Prevention B.V. 

Cotton rat studies were conducted at Sigmovir Biosystems, Inc. by permission of the Institutional Animal Care and Use Committee (IACUC) of Sigmovir Biosystems, Inc.

The AGM study was conducted at the Wake Forest School of Medicine test facility and approved by the IACUC of Wake Forest University (WFU). 

### 2.2. Vaccines

Ad26.RSV.preF and preF protein vaccines used in this study have previously been described in detail [22,23]. 

### 2.3. RSV Strains

Various RSV A and RSV B strains, including clinical isolates, were used for RSV pre-exposure, in virus neutralization assays, and as challenge strains in protective efficacy studies. A phylogenetic tree based on the F protein sequence, visualizing their relationship, is displayed in Supplementary Figure S3. RSV A and RSV B clinical isolates used in this study were isolated in the Netherlands. Further characteristics of the virus strains are detailed in Supplementary Table S1. 

### 2.4. In Vivo Study Description: Immunogenicity Assessment in Naïve and RSV Pre-Exposed Mice

Twelve weeks prior to immunization, BALB/c mice (Charles River Laboratories; 6–8 weeks old) were intranasally pre-exposed with 5 × 10^5^ plaque-forming units (pfu) of RSV A 18-001989 or RSV B 17-058221. At week 12, groups of age-matched naïve mice were added to the study, and mice were immunized intramuscularly (i.m.) with 10^8^ vp Ad26.RSV.preF (10^9^ vp for the naïve groups), or with identical doses of Ad26 vector without transgene insert (mock). Serum samples were collected for assessments of humoral responses. 

### 2.5. In Vivo Study Description: Immunogenicity and Protective Efficacy Assessment in Cotton Rats

Female cotton rats (Sigmovir Biosystems, Inc., Rockville, MD, USA; 6–8 weeks old) were immunized intramuscularly at Week 0 with the indicated doses of Ad26.RSV.preF, or at Week 0 and Week 4 with preF protein, after which animals were challenged at Week 7. The challenge was conducted by intranasal application of RSV A2, RSV A Memphis, RSV A Long, or RSV B Wash 18537 (all 1 × 10^5^ pfu per animal) or RSV B 17-058221 (1 × 10^4^ pfu per animal). Note, the challenge dose for RSV B 17-058221 was optimized at 1 × 10^4^ pfu, as this resulted in a similar lung and nose viral load as the 1 × 10^5^ pfu challenge dose, but with more robust responses in a pilot study. Animals were sacrificed 5 days post-challenge, and the viral load was determined by plaque assay. Serum samples were collected prior to the challenge for the readout of humoral immune responses. Cotton rat experiments were performed by Sigmovir Biosystems, Inc.

For passive transfer studies, young cotton rats (4 weeks of age) received an intraperitoneal injection with 2 mL of (diluted) serum, 10 mg/kg of Synagis, or PBS, one day prior to the challenge that was performed as described above. For human serum transfer studies, a serum pool was generated from serum samples of 39 randomly selected human participants (age 65 years and older) that were intramuscularly immunized with a mixture of 10^11^ vp Ad26.RSV.preF and 150 μg preF protein, isolated pre-vaccination and at 14 days post-vaccination (Clinicaltrials.gov ID NCT03982199).

### 2.6. In Vivo Study Description: Immunogenicity in African Green Monkeys

A detailed description of the study in African Green Monkeys is given by Saeland et al. [23]. In short, adult and elderly female animals (9–26 years of age) were pre-exposed with 7.5 × 10^5^ pfu RSV Memphis at 19 weeks prior to intramuscular immunization at Week 0 with 10^11^ vp Ad26.RSV.preF, 150 µg preF protein or a combination of both. Serum isolated from 4 animals of each group at Week −5, Week 2 and Week 55 was analyzed for neutralizing antibody titers against a panel for RSV A and RSV B clinical isolates.

### 2.7. Microneutralization Assay

Microneutralization antibody titers were determined using RSV-susceptible Vero cells. Heat-inactivated serum samples were serially diluted and mixed with 1000 pfu of the different RSV strains. After 1 h incubation at room temperature, Vero cells were added, and the plates were incubated for 4 days at 37 °C. The monolayers were washed and fixed with 80% cold acetone. RSV replication was determined by F protein expression using a biotin-conjugated anti-F monoclonal antibody MAb8262 (clone 133-1H, Merck, Darmstadt, Germany), which binds to RSV A or RSV B F protein. Plates were incubated with streptavidin-horseradish peroxidase, and after washing, a chemiluminescent substrate (LumiGLO, SeraCare, Milford, MA, USA) was added. The luminescence signal was determined with the Biotek Synergy Neo plate reader. Inhibitory concentration 50% (IC50) titers were calculated.

### 2.8. ELISA

ELISA was used to measure IgG antibodies binding to preF proteins derived from RSV A or RSV B sequences. Briefly, 96-well plates were coated with streptavidin (0.66 µg/mL) and incubated at 4 °C overnight. Wells were washed and then blocked with bovine serum albumin for 30 min at RT. After washing, biotinylated RSV preF protein (0.3125 µg/mL) was added and incubated for 1 h at RT. The wells were washed again, and serially diluted heat-inactivated serum samples and standards were added to the wells and incubated for 1 to 2 h at RT. RSV preF–specific antibodies were detected by horseradish peroxidase (HRP)–labeled anti-cotton rat IgG (Southern Biotech, Birmingham, AL, USA). The reaction was developed with LumiGLO^®^ substrate (SeraCare, Milford, MA, USA), and the luminescence signal was measured at 428 nm. Titers were expressed as a log_10_ relative potency in comparison with a standard serum sample. 

### 2.9. Plaque Titration Assay for RSV Titers in Cotton Rat Challenge Model

Lung and nasal tissues were obtained 5 days after the challenge. Left lung lobes from each cotton rat were weighed prior to homogenization; nasal tissue samples were estimated to be 0.3 g. Plaque titration was performed in duplicates for the homogenized tissue samples on HEp-2 cells in a 24-wells format. Cells and homogenate were incubated for 2 h, the supernatant was aspirated, and wells were overlayed with methylcellulose media until plaque formation (4 to 7 days, depending on the RSV strain used). Plates were stained with crystal violet, and plaques were counted to determine the virus concentration in pfu/g of tissue.

## 3. Results

### 3.1. Ad26.RSV.preF Induced Broadly Neutralizing Antibodies against RSV A and RSV B Strains in Naïve and RSV Pre-Exposed Rodents

**Figure 1:**
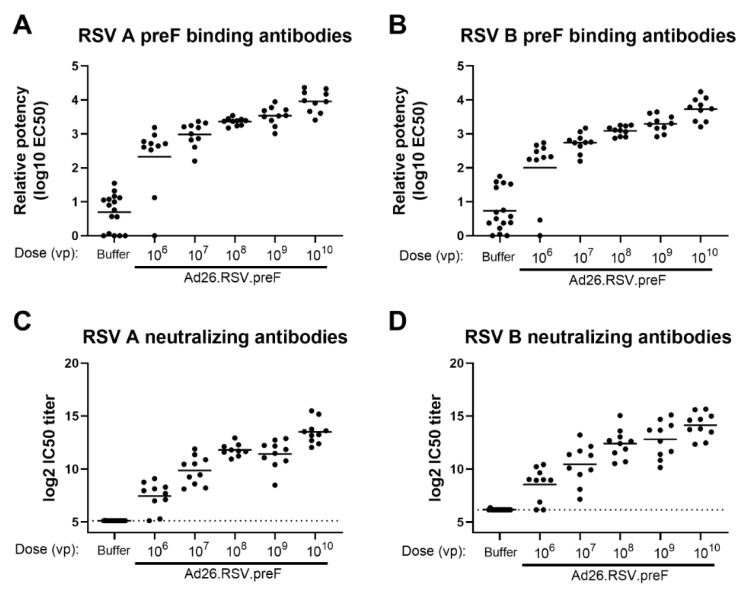
Ad26.RSV.preF dose-dependently induces antibodies binding to preF from RSV A and RSV B, which are capable of neutralizing both RSV A and RSV B strains in naïve cotton rats. Cotton rats were immunized with 10^6^ to 10^10^ vp Ad26.RSV.preF (n = 10 per group) or formulation buffer (n = 16) at day 0, and serum isolated at day 49 was analyzed. Binding IgG was determined by ELISA using RSV A preF (**A**) or RSV B preF (**B**). Neutralizing antibody titers were determined against RSV A 18-001989 (**C**) or RSV B 17-058221 (**D**) by microneutralization virus neutralization assay (MN VNA). The lower limit of detection for MN VNA was defined as the mean plus 3× the standard deviation of the formulation buffer group, indicated with a dotted line. Means per group are indicated with horizontal lines. Note: MN VNA titers against RSV A 18-001989 from one animal in the 10^8^ vp dose group were unexpectedly and unexplainably high (24.2 log2 IC50), and this data point was excluded.

The ability of RSV A2-based preF, encoded by the Adenoviral vector Ad26, to induce antibodies that are directed against both RSV A and RSV B subtypes was assessed in naïve cotton rats. Animals received a single immunization with increasing doses of Ad26.RSV.preF, ranging from 10^6^ to 10^10^ viral particles (vp). A dose-dependent increase in antibodies binding to RSV A and RSV B-derived preF was observed (Figure 1A,B), which were capable of neutralizing recent clinical isolates of RSV A 18-001989 and RSV B 17-058221 (Figure 1C,D) (*p* < 0.0001 for all parameters assessed, tested using the non-parametric dose trend Jonckheere-Terpstra Test). Seroconversion for both assay viruses was already observed in the majority of animals receiving the lowest vaccine doses. 

**Figure 2:**
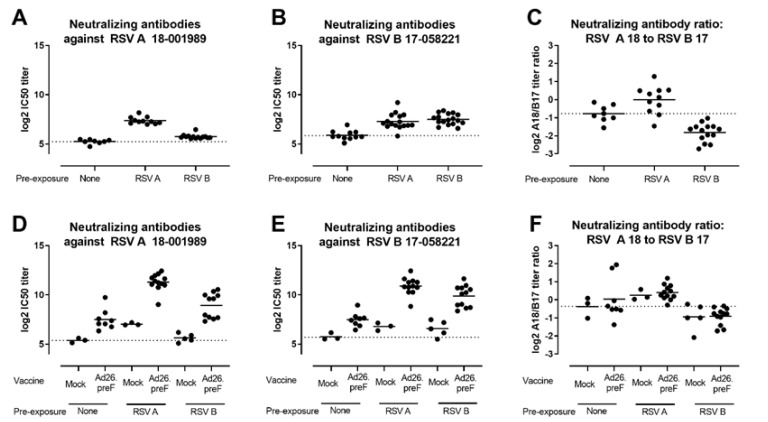
Ad26.RSV.preF induces RSV A and RSV B subtype-specific neutralizing antibodies in both RSV A and RSV B pre-exposed mice. Mice were pre-exposed to RSV A 18-001989 or RSV B 17-058221 12 weeks prior to immunization with 10^8^ vp Ad26.RSV.preF (n = 12), or empty Ad26 vector (mock, n = 5). As controls, naïve mice were immunized with 10^9^ vp Ad26.RSV.preF (n = 8), or empty Ad26 vector (mock, n = 3). Neutralizing antibodies titers against RSV A 18-001989 (**A**,**D**) or RSV B 17-058221 (**B**,**E**) were determined by MN VNA on serum isolated at week 12, prior to immunization (**a**,**b**), and at week 16, 4 weeks posts immunization (**D**,**E**). Log2 ratios between RSV A and RSV B VNT are depicted (**C**,**F**). The average responses in mock immunized naïve animals are indicated with dotted lines. Mean responses per group are indicated with horizontal lines. Note some data points are missing due to insufficient serum availability, dropout of animals from the study or experimental failure.

To reflect the human situation better, where all adults have been exposed to RSV, the elicitation of RSV A and RSV B-neutralizing antibodies by Ad26.RSV.preF was additionally assessed in an RSV pre-exposure mouse model. Mice were pre-exposed to RSV A or RSV B by intranasal administration of live RSV and immunized 12 weeks later with 10^8^ vp Ad26.RSV.preF. As controls, naïve mice were immunized with a tenfold higher Ad26 dose. Pre-exposure to either RSV A or RSV B strains induced both RSV A and RSV B neutralizing antibodies when measured prior to immunization (Figure 2A,B). Clear skewing of the response by the pre-exposure virus was demonstrated by assessing the ratio between RSV A and RSV B virus-neutralizing antibody titers (VNT) prior to immunization (Figure 2C). Ad26.RSV.preF immunization boosted the neutralizing antibody titers against both RSV subtypes in both RSV A and RSV B pre-exposed animals (Figure 2D,E). Interestingly, the skewing of the virus-neutralizing antibody responses induced by the pre-exposure with RSV A or RSV B did not change after immunization with Ad26.RSV.preF (Figure 2F), indicating that the vaccine boosted pre-existing RSV A and RSV B-directed responses to a similar extent. 

### 3.2. Immunization with Ad26.RSV.preF Induced Protection against Challenge with RSV A and RSV B Strains in Naïve Cotton Rats

To assess the protective efficacy of the Ad26.RSV.preF vector, various RSV A and RSV B cotton rat challenge models were developed (RSV A2, RSV A Long, RSV A Memphis, RSV B Wash 18537 and RSV B 17-058221). In order to validate these different models, the efficacy of the monoclonal antibody Palivizumab was assessed. It has been demonstrated earlier that Palivizumab showed equal virus-neutralizing capacity against RSV A and RSV B strains, as well as equal protective capacity in cotton rats challenged with RSV A or RSV B strains [15]. For all RSV A and RSV B strains tested, prophylaxis with Palivizumab at a dose of 10 mg/kg provided complete protection in the lungs and a reduction of viral load in the nose (Supplementary Figure S1). 

**Figure 3:**
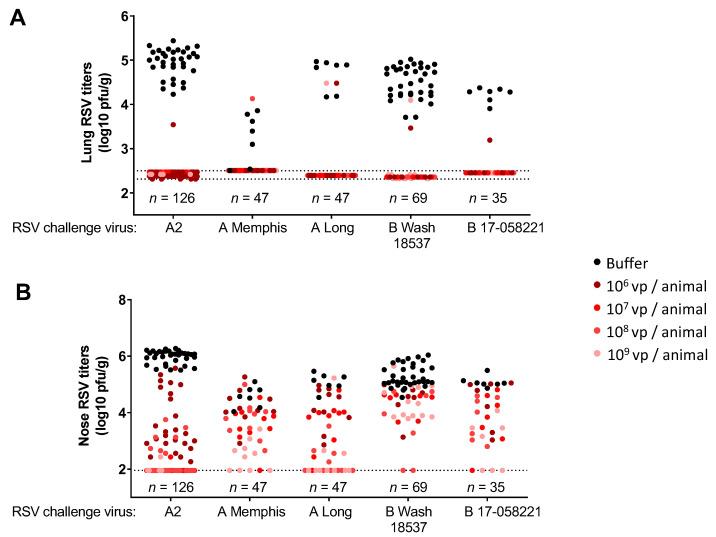
Immunization with Ad26.RSV.preF confers protection against challenges with RSV A and RSV B strains in naïve cotton rats. Cotton rats were immunized with 10^6^ to 10^9^ vp of Ad26.RSV.preF at day 0, and challenged with RSV A2, RSV A Memphis, RSV A Long, RSV B Wash 18537 or RSV B 17-058221 at week 7. RSV viral load was determined by plaque assay in homogenates of the lung (**A**) or nose tissue (**B**) isolated at day 5 post-challenge and expressed as log10 plaque forming units (pfu) per gram of tissue. Compiled results from multiple challenge studies are shown (five studies for RSV A2, one study for RSV A Memphis, one study for RSV A Long, four studies for RSV B Wash 18537 and one study for RSV B 17-058221), with total numbers of animals per challenge strain shown in the figure, and number of animals per Ad26.RSV.preF dose level (indicated with different colored dots) ranging from seven to forty-two. Pooling of results of several different studies is considered justified, as viral loads in the control groups were within acceptable ranges between the separate studies that use the same challenge strain. The limit of detection of the plaque assays is indicated with dotted lines.

We assessed the protective efficacy of Ad26.RSV.preF against RSV A and RSV B strains, using the various cotton rat challenge models. A single immunization with Ad26.RSV.preF provided good protection against both RSV A and RSV B. For all the RSV A and RSV B strains tested, (almost) complete lung protection was achieved at all vaccine doses tested (10^6^ vp to 10^9^ vp), with only a very few animals showing breakthrough infections (Figure 3A). Vaccine dose-dependent reduction of nose viral load was observed for all RSV strains tested, except for RSV B Wash 18537, where nose protection seemed relatively poor (Figure 3B). 

Similar studies were conducted where cotton rats were immunized with increasing doses of unadjuvanted soluble preF protein. Results were highly similar to Ad26.RSV.preF, i.e., preF protein-induced antibodies were capable of neutralizing recent clinical isolates of RSV A and RSV B strains, with largely similar titers observed against both strains. In addition, preF protein provided protective efficacy against the challenge with RSV A2, RSV B Wash 18537 and RSV B 17-058221 strains (Supplementary Figure S2).

### 3.3. Ad26.RSV.preF and the Combination of Ad26.RSV.preF and preF Protein Induce Durable RSV A and RSV B Cross-Neutralizing Antibodies in RSV-Pre-Exposed African Green Monkeys

We demonstrated earlier that in African Green Monkeys (AGMs) that were pre-exposed to RSV A Memphis prior to immunization, Ad26.RSV.preF and the combination of Ad26.RSV.preF and RSV preF protein induced high and durable VNT against RSV A CL57, whereas the induced titers declined more rapidly after immunization with RSV preF protein alone [23]. 

Here, we assessed the breadth of the serum-neutralizing antibodies induced in these AGMs by MN VNA using six different RSV clinical isolates. We selected three RSV A strains and three RSV B strains from an RSV strain collection isolated from the 2011/2012 to 2017/2018 seasons in the Netherlands. Strains were selected based on coverage of the isolation period and their genetic diversity of the nucleotide sequence of the F protein (Supplementary Figure S3). 

**Figure 4:**
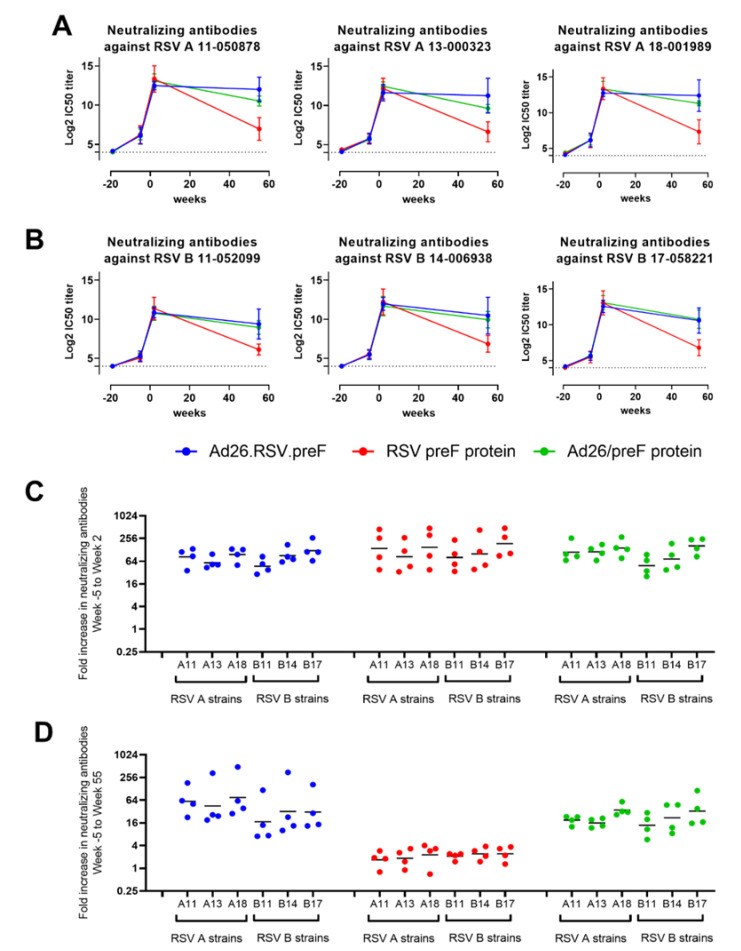
Ad26.RSV.preF, RSV preF protein and their combination induce antibodies that can neutralize various RSV A and RSV B strains in RSV A Memphis pre-exposed African Green Monkeys. African Green Monkeys were intranasally pre-exposed with RSV A Memphis 37 at week −19 and immunized intramuscularly at Week 0 with 10^11^ vp Ad26.RSV.preF (in blue), 150 μg preF protein (in red), or a mixture of both components (in green). MN VNT against 3 RSV A strains (**A**) and 3 RSV B strains (**B**) were determined in serum samples (n = 4 per group at week −5, week 2 and week 55; one sample per group at week −19). The 50% inhibitory concentration (IC50) titers were calculated, and the limit of detection (LOD) was set at 4 log2 (dotted line), which is the lowest serum dilution used in the assay. Mean ± standard deviations are shown. Fold increase from week −5 to week 2 (**C**) or to week 55 (**D**) was calculated per animal, with geometric means per group per RSV strain indicated.

Pre-exposure to RSV A Memphis induced VNT against all RSV A and RSV B strains tested, as evidenced by seroconversion in all animals at the pre-immunization time point (week −5). VNT against all strains were strongly and similarly increased after immunization with Ad26.RSV.preF, RSV preF protein or the combination of both when measured 2 weeks after immunization. Durability was demonstrated after immunization with Ad26.RSV.preF and the mix, as high VNT against all RSV A and RSV B strains tested, was still detectable 55 weeks after a single immunization. In contrast, titers induced by the RSV preF protein declined more rapidly for all strains, although these were still detectable at week 55 (Figure 4A,B). 

In addition, the increase in VNT per animal after immunization compared to pre-immunization titers was largely similar for the RSV A and RSV B strains tested (Figure 4C,D).

### 3.4. Antibodies Induced by the Combination of Ad26.RSV.preF and preF Protein in Human Subjects Provide Protection against RSV A and RSV B Challenge in the Cotton Rat Challenge Model

The combination of Ad26.RSV.preF and RSV preF protein was evaluated in a phase 1/2a clinical study in adults aged 60 years and above (Clinicaltrials.gov ID NCT03502707). Antibodies capable of neutralizing various RSV A and RSV B strains, including 12 RSV clinical strains (6 RSV A strains and 6 RSV B strains), were induced 29 days post-immunization, with similar increases in titers for RSV A and RSV B subtypes (Comeaux et al., submitted).

**Figure 5:**
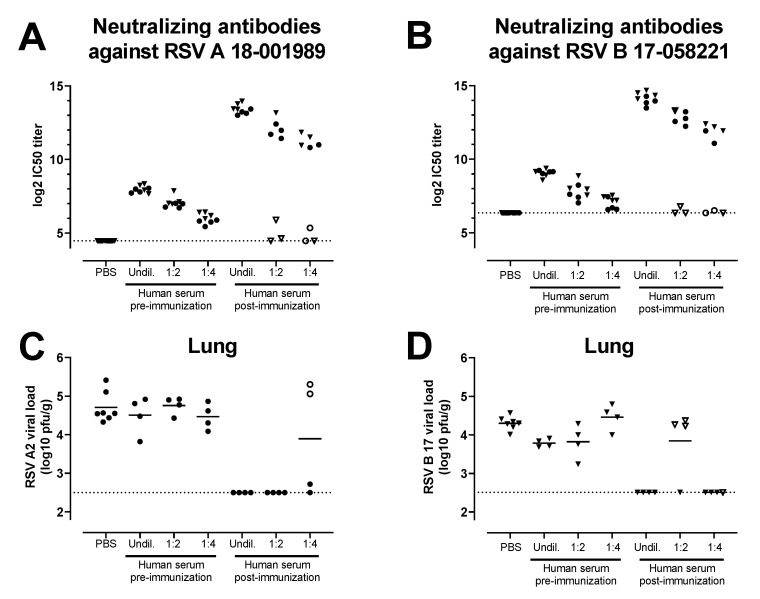

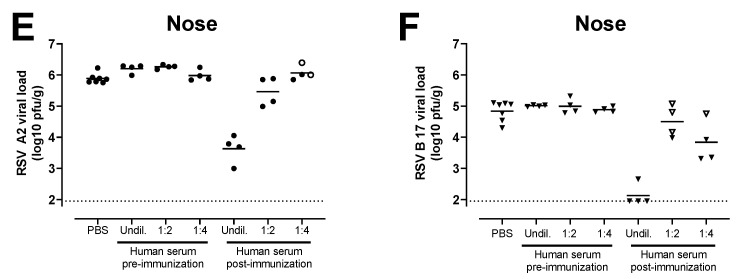
Serum of human subjects that received vaccination with the combination of Ad26.RSV.preF and RSV preF protein can provide protection against RSV A and RSV B strains in the cotton rat model. Cotton rats received different amounts (2 mL of undiluted (undil.), 1:2 or 1:4 diluted serum in PBS) of a human serum pool generated by pooling serum isolated pre-vaccination or at 14 days post-vaccination with a mixture of 10^11^ vp Ad26.RSV.preF and 150 μg preF protein (n = 39) by intraperitoneal injection (n = 4 per group) or with PBS (n = 7). The next day, pre-challenge serum samples of the recipient animals were isolated and assayed for VNT against RSV A 18-001989 (**A**) or for RSV B 17-058221 (**B**). Animals were challenged with RSV A2 (**C**,**E**) or RSV B 17-058221 (**D**,**F**). RSV viral load was determined five days after the challenge using plaque assay on homogenates of the lung (**C**,**D**) or nose tissue (**E**,**F**) and expressed as pfu per gram of tissue. Samples of animals that were (to be) challenged with RSV A2 are indicated with dots, whereas samples of animals that were (to be) challenged with RSV B 17-058221 are indicated with triangles. Animals in which serum transfer was not successful, based on low VNT pre-challenge, are indicated with open symbols. LODs for MN VNA were determined as the mean plus 3× standard deviations of the PBS control group. LODs are indicated with dotted lines.

Sera from a clinical study that assessed the same Ad26.RSV.preF and RSV preF protein combination vaccine in adults aged 65 years and above (NCT03982199) were tested for the ability to induce protective efficacy against RSV A and RSV B in serum transfer cotton rat challenge studies. A pool was generated of sera isolated pre-vaccination and at 14 days post-vaccination with Ad26.RSV.preF/preF protein mix from 39 human subjects. Serial dilutions of these human serum pools were transferred intraperitoneally into cotton rats. MN VNA on pre-challenge serum of the cotton rats verified the successful transfer of the antibodies into the majority of the cotton rats, with clear serum dose-dependent titers in the recipient animals (Figure 5A,B). Animals were challenged one day after the serum transfer with either RSV A2 or RSV B 17-058221. Limited or no protection against RSV infection in the lungs and nose was observed with the serum pool isolated pre-vaccination. In contrast, (almost) complete protection in the lung against RSV A2 or RSV B 17-058221 challenge was observed in animals with successful transfer of post-vaccination serum at all the serum doses applied (Figure 5C,D). In addition, a serum dose-dependent reduction of nose viral load was observed for the animals challenged with RSV A2 or RSV B 17-058221 (Figure 5E,F).

## 4. Discussion

Our data demonstrate that antibodies induced by monovalent RSV A-based vaccine components Ad26.RSV.preF, preF protein, or a combination of those can neutralize strains of both RSV A and RSV B subtypes, including contemporary strains. This was demonstrated in naïve rodents as well as in RSV pre-exposed mice and nonhuman primates, where vaccination did not alter the RSV A or RSV B skewing induced by the RSV pre-exposure. Protective efficacy against RSV A and RSV B strains was observed in the cotton rat challenge model after immunization with Ad26.RSV.preF or preF protein, with good protection of the lower respiratory tract and various degrees of upper respiratory tract protection. No clear distinction between the level of upper respiratory tract protection and the RSV subtype was observed. Therefore, the small differences observed may be linked to the stringency of the different challenge models rather than to the RSV subtype. Importantly, data in this manuscript demonstrated that serum from humans immunized with the combination of Ad26.RSV.preF and preF protein can confer protection against RSV A and RSV B strains in the cotton rat challenge model, suggesting the RSV A-based vaccine is likely to confer clinical efficacy against both RSV A and RSV B subtypes.

Subdivision of the RSV A and RSV B antigenic groups was originally done through the use of monoclonal antibodies and cDNA probes on several circulating strains in 1985. Deep sequencing approaches subsequently helped to broader define RSV diversity, although still only a relatively limited number of sequence data is publicly available. Up to now, 37 genotypes have been identified for RSV B and 13 for RSV A, mainly based on genetic variation in the G glycoprotein (reviewed in [25]). It was reported that the evolutionary rate for both subtypes differs from each other, with a lower rate of nucleotide substitutions/site/year for RSV A than RSV B [26]. Other studies showed that RSV B has a lower consensus diversity than RSV A at the population level, while exhibiting greater within-host diversity [27]. Whereas RSV diversity and evolution are predominantly found in the G glycoprotein [26], evolutionary changes in the F protein are also found. Interestingly, in recent years the F protein of RSV B has shown higher sequence variation than the F protein from RSV A, and this is mainly observed at important antigenic sites of RSV B F [26]. This underscores the importance of continued genetic surveillance to monitor the clinical effectiveness of immunoprophylactic products, both MAbs and vaccines.

RSV A subtype specificity of RSV F-directed monoclonal antibodies was described earlier, for example, for the RSV preF directed MAb D25 [17,18], although this was not confirmed in other studies [19], or using the D25 derivative Nirsevimab [15]. These discrepancies can be due to specific subtle differences in the RSV B strains and antigens used in the different studies. Indeed, it was demonstrated that a specific amino acid alteration in the F protein (K68N) is associated with reduced susceptibility to Nirsevimab [28] and that this alteration can be found in RSV B consensus sequences [27]. In addition, only two mutations in the F protein in circulating RSV B strains at positions 172 and 173 caused a lack of clinical efficacy of Suptavumab against RSV B [20]. Also, after active vaccination with the preF protein DS-Cav1, RSV subtype-specific skewing was suggested. Immunization of nonhuman primates or humans with this RSV A-based protein resulted in a higher increase in titers against a recombinant RSV strain expressing the F gene from RSV A2 compared to a strain expressing F from RSV B 18537 [11,21]. Interestingly, this RSV B strain appeared to be the most stringent in our challenge studies as it was the most difficult to induce protection against, whereas protection against a recent clinical RSV B strain appeared similar to the different RSV A strains tested (Figure 3B). This underscores the necessity to test multiple strains, including strains representative of currently circulating RSV A and RSV B strains, to draw conclusions on the subtype specificity of the responses induced. The results shown in this paper using the RSV A-based Ad26.RSV.preF and preF protein demonstrate similar levels of protection and neutralization against a panel of different RSV A and RSV B strains, including recent clinical isolates, and indicate no subtype-specific skewing. This is in line with results from a recent clinical study in older human subjects immunized with the Ad26/preF protein combination that showed similar cross-neutralization against a panel of 12 RSV clinical strains (six RSV A strains and six RSV B strains) (Comeaux et al., submitted), including the same RSV strains used in the preclinical studies described in this manuscript. 

The term ‘original antigenic sin’ (OAS) describes how one’s first exposure to an infection shapes the outcome of subsequent exposures to antigenically related strains by preferentially employing immunological memory [29]. The manifestation of this phenomenon depends, among other factors, on the relatedness of the antigens between the different infections or vaccines and has been studied extensively in the context of influenza [30]. Whereas for influenza the antigenic difference between circulating and drifting strains can be quite big, indications for OAS have also been observed for more closely related antigens, for example, for emerging SARS-CoV-2 variants. Subjects who were vaccinated with a Wuhan spike-containing vaccine and later infected with the Omicron variant did not develop unique Omicron-specific antibodies but rather re-activated memory B cells recognizing conserved epitopes (reviewed by [31]). Similar results were observed in nonhuman primates given a prime vaccination with mRNA-1273 (containing the Wuhan spike), followed by an mRNA-Omicron boost vaccination [32]. Literature on OAS in the context of RSV infections is sparse. However, this mechanism may be relevant, as almost all infants are exposed to RSV before two years of age and undergo re-infections throughout life. In a recent paper, it was shown that induction of neutralizing antibody responses against both RSV A and RSV B subtypes after an RSV infection were greater against prototypic strains compared with their contemporaneous counterparts [33], indicating that OAS may also occur in the context of RSV. Our own results in RSV pre-exposed mice also support the concept of OAS. The skewing of the neutralizing antibody response towards either RSV A or RSV B, which is induced by pre-exposure (Figure 2C), is maintained after immunization with RSV A-based Ad26.RSV.preF (Figure 2F). This suggests that the cross-reactive memory repertoire that is initially induced by the pre-exposure, is boosted by the vaccine irrespective of the RSV subtype of the vaccine antigen insert. 

Passive transfer of serum from humans immunized with the combination of Ad26.RSV.preF and preF protein in cotton rats elicited full protection in the lower respiratory tract and a more limited reduction of viral load in the upper respiratory tract for both RSV A2 and RSV B 17-058221 challenge viruses (Figure 5). Similar results were observed with 10 mg/kg of the monoclonal antibody Palivizumab, which provided complete protection in the lung against RSV A2, RSV A Memphis, RSV A Long, RSV B Wash 18537 and RSV B 17-058221, whereas in the nose, a reduction of viral load was observed for all strains rather than complete protection (Suppl Figure S1). These results are in line with earlier publications showing complete protection in the lung against RSV A2 and RSV B9320 and only marginal reduction of nose viral load after prophylaxis with 8 mg/kg Palivizumab [15]. From these data, it can be concluded that full protection of the lower respiratory tract in the cotton rat model can be achieved by (neutralizing) antibodies, whereas protection of the upper respiratory tract is harder to achieve by systemically delivered antibodies. Antibody diffusion into the upper respiratory tract might be less efficient when compared to the lower respiratory tract, thereby (partly) explaining the difference in the level of protection in the upper and lower respiratory tracts induced by passive immunization, as well as by active immunization (Figure 3). 

Our results collectively demonstrate that the RSV A-based Ad26.RSV.preF, preF protein and the combination of both can induce neutralizing antibodies and protection against both RSV A and RSV B subtypes without apparent subtype specificity. This implies that these vaccine candidates can confer protective clinical efficacy against both RSV subtypes.

## Data Availability

All data to understand and assess the conclusions of this research are available in the main text and supplemental materials. The raw data that support the findings of this study are available from the corresponding author upon reasonable request.

## Supplementary Materials

The following supporting information can be downloaded at: https://www.mdpi.com/article/10.3390/vaccines11030672/s1. Figure S1: Setting up RSV A and B challenge models in cotton rats using Palivizumab to assess prophylactic efficacy. Figure S2: RSV preF protein induces across RSV subtype immunogenicity and protective efficacy in naïve cotton rats. Figure S3: Phylogenetic tree visualizing the evolutionary relationships of the F protein used in the various assays. Table S1: Characteristics of RSV virus stocks used.
