# Supplementary material for: RSV A2-Based Prefusion F Vaccine Candidates Induce RSV A and RSV B Cross Binding and Neutralizing Antibodies and Provide Protection against RSV A and RSV B Challenge in Preclinical Models
Source: Vaccines (Basel). 2023 Mar 16;11(3):672. doi: 10.3390/vaccines11030672 (PMC10057437; doi:10.3390/vaccines11030672)
Supplement: Supplementary file 1 [file vaccines-11-00672-s001.zip › vaccines-2244358-supplementary.pdf]

Supplementary Materials

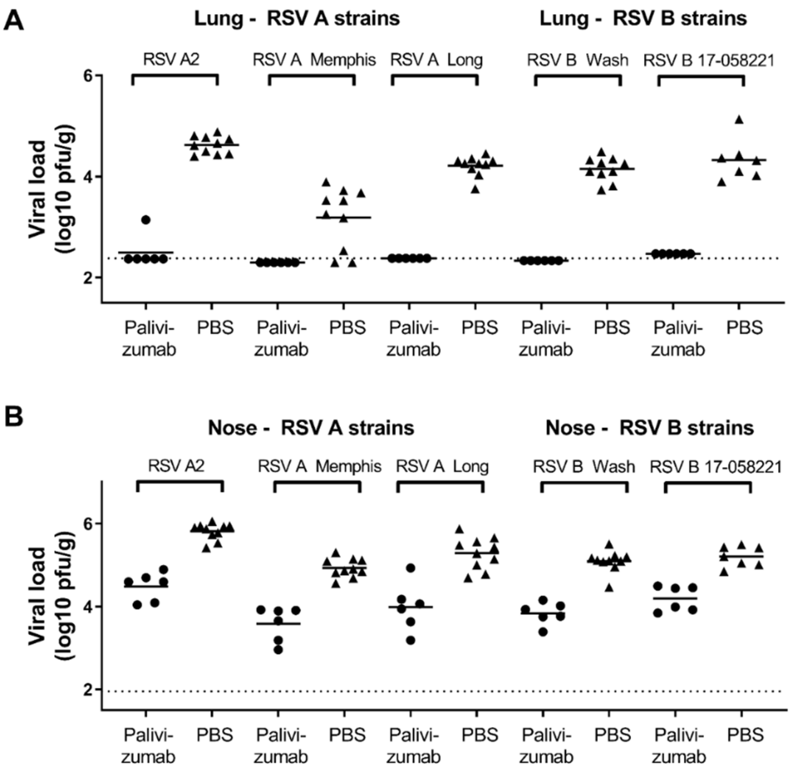

Supplementary Figure S1. Setting up RSV A and B challenge models in cotton rats using Palivizumab to assess prophylactic efficacy. Animals were injected intraperitoneally with 10 mg/kg Palivizumab (n = 6 per group), or PBS (n = 10 or n = 7 per group), and challenged one day later with RSV A2, RSV A Memphis, RSV A Long, RSV B Wash 18537 or RSV B 17-058221. RSV viral load was determined 5 days after challenge using plaque assay on homogenates of lung (**a**) or nose tissue (**b**), and expressed as pfu per gram of tissue. Lower limits of detection are indicated with dotted lines.

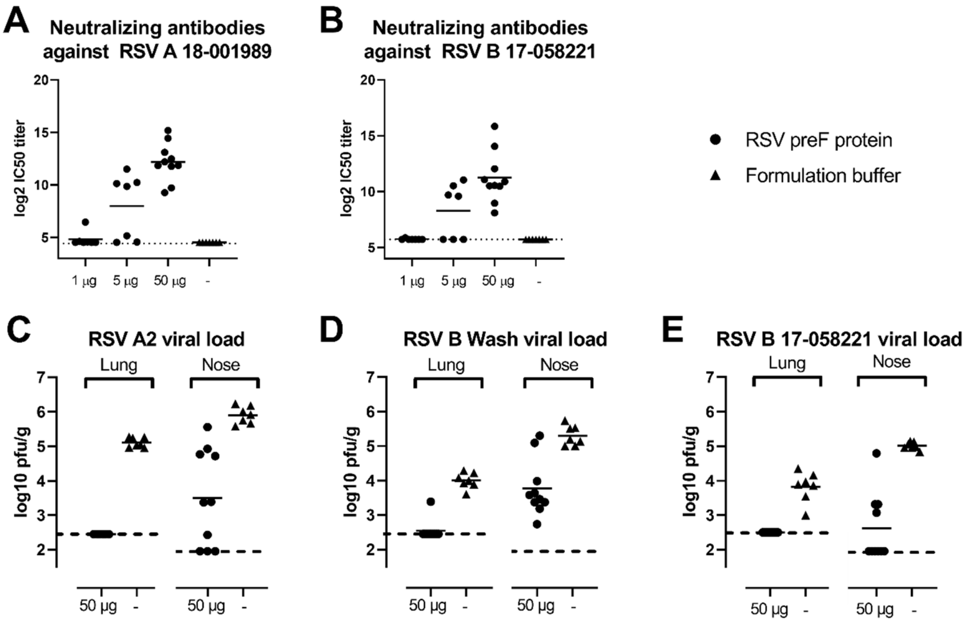

Supplementary Figure S2. RSV preF protein induces across RSV subtype immunogenicity and protective efficacy in naïve cotton rats. Cotton rats were immunized at day 0 and day 28 with 1, 5 (n = 7 per group) or 50 µg (n = 10) of RSV preF protein. Control animals received formulation buffer (n = 7). Serum was isolated at day 49 and analysed by MN VNA for neutralizing antibodies against RSV A 18-001989 (**a**) or RSV B 17-058221 (**b**). Cotton rats were immunized with 50 µg RSV preF protein at day 0 and day 28 and subsequently challenged with RSV A2 (**c**), RSV B Wash 18537 (**d**) or RSV B 17-058221 (**e**) at day 49. RSV viral load was determined 5 days post challenge by plaque assay in homogenates of lung or nose tissue, and expressed as pfu per gram of tissue. Group means are indicated with horizontal lines, and lower limits of detection are indicated with dotted or dashed lines.

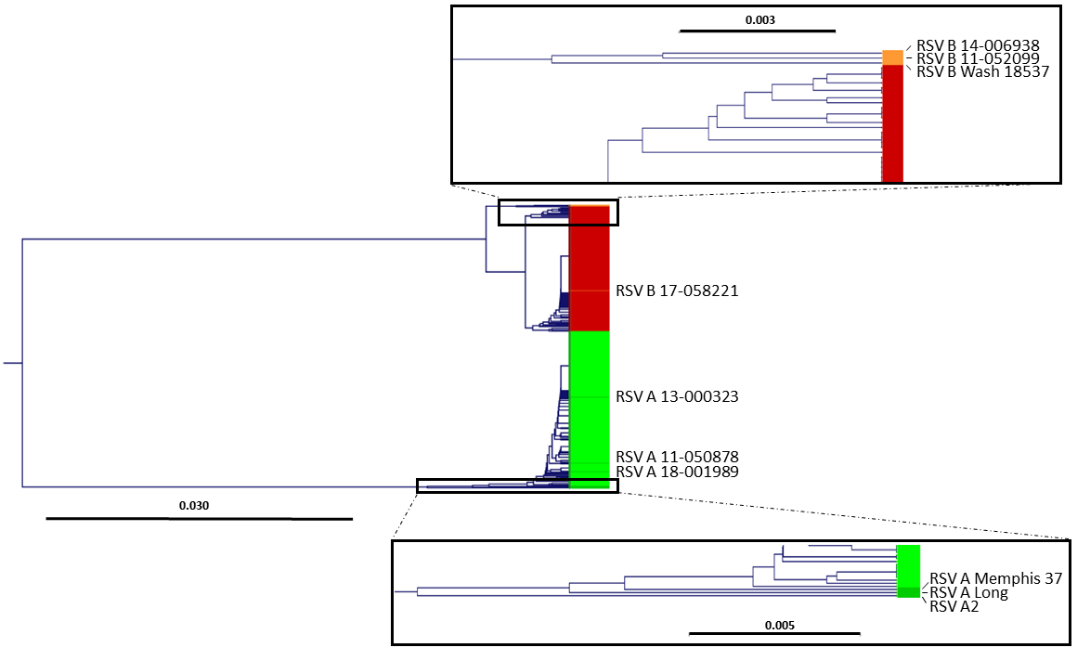

Supplementary Figure S3. Phylogenetic tree visualizing the evolutionary relationships of the F protein used in the various assays. Display of relationship between the protein sequences of RSV F between the RSV strains used for analysis of neutralizing antibodies and protective efficacy, together with a collection of publicly available F sequences from global RSV A (indicated in green) and RSV B (indicated in red) strains isolated from 2018 to Jul-2021 (n = 462). Protein sequences and meta data were obtained from the public database ViPR (Virus Pathogen Resource: https://www.viprbrc.org). The scale bars represent the percentages of different amino acids. Figure generated using CLC Main Workbench v22.0 (Qiagen Aarhus A/S). Note: sequence information for the F protein for some of the lab-adapted strains is not complete (missing 7 amino acids (RSV A Long, RSV A2), or 16 amino acids (RSV A Memphis 37)), thereby potentially resulting in minor imprecisions in the phylogenic tree.

Supplementary Table S1: Characteristics of RSV virus stocks used

RSV strain	Year of isolation	Isolated from	Cell line used for propagation	Infectious titer stock (pfu/mL)	Passage used
RSV A2	1961	Lower respiratory tract	HEp-2	$3x10^8$	Not known
RSV A Memphis	2006	Respiratory secretion	HEp-2	$1.9x10^7$	Not known
RSV A Long	1956	Not specified	HEp-2	$5x10^7$	Not known
RSV B Wash 18537	1962	Respiratory secretion	MA-104 / HEp-2	$5x10^7$ / $4x10^6$	Not known
RSV A 11-050878	2011	Nasopharyngeal lavage	HEp-2	$3.9x10^7$	p2
RSV A 13-000323	2013	Sputum	HEp-2	$2.2x10^7$	p2
RSV A 18-001989	2018	Sputum	HEp-2	$1.3x10^7$ / $8.0x10^6$	p2 / p3
RSV B 11-052099	2011	Nasopharyngeal lavage	HEp-2	$1.3x10^6$	p2
RSV B 14-006938	2014	Throat swab	HEp-2	$1.3x10^7$	p2
RSV B 17-058221	2017	Sputum	HEp-2	$1.3x10^7$ / $5.3x10^6$ / $1.3\ x10^7$	p2 / p3 / p3
